# Supplementary material for: Content-rich biological network constructed by mining PubMed abstracts
Source: BMC Bioinformatics. 2004 Oct 8;5:147. doi: 10.1186/1471-2105-5-147 (PMC528731; doi:10.1186/1471-2105-5-147)
Supplement: Additional File 2 — The original results of the above study (non-essential files are deleted to keep the file size under the limit set by BMC bioinformatics). [file 1471-2105-5-147-S2.bz2 › chilibotAdditionalFile2/dip30/484ID8702798E10575/html/IL8RA_PPBP.html]

 


 **IL8RA** and **PPBP** 
  
Found 25 abstracts in PubMed, retrieved 25.  
 

 What does Google say? 
 PDF only 
| .edu only 

---

**Interactive relationship** (e.g. stimulation, inhibition, etc)

**Neutral relationship**- These results provide evidence that both receptors are involved in NAP 2  [ **PPBP** ]  induced neutrophil chemotaxis, with CXCR  [ **IL8RA** ]  2 rendering the cells responsive to low dosages of the chemokine, and with CXCR 1  [ **IL8RA** ]  extending their responsiveness to NAP 2  [ **PPBP** ]  dosages higher by several orders of magnitude.  Ref: 9373270 Blood, 1997
- ... the interleukin 8 receptor type 2 IL8R2 previously designated  **IL8RA**  also binds growth regulated gene GRO, and neutrophil activating protein 2 NAP 2  [ **PPBP** ]  with high affinity.  Ref: 1427896 Genomics, 1992
- Immunoprecipitation and Western blot analyses of surface expressed receptors covalently linked to IL 8 or NAP 2  [ **PPBP** ]  as well as in their unloaded state revealed the occurrence of a single CXCR  [ **IL8RA** ]  2 variant with an apparent size of 56 kDa.  Ref: 10878382 J Immunol, 2000
- Similar to other members of this group, such as IL 8, NAP 2  [ **PPBP** ]  activates chemotaxis and degranulation in neutrophils polymorphonuclear PMN through chemokine receptors CXCR 1  [ **IL8RA** ]  and CXCR  [ **IL8RA** ]  2.  Ref: 11049972 Blood, 2000
- **IL8RA**  is a promiscuous receptor for at least two other related ligands, GRO alpha and NAP 2  [ **PPBP** ] .  Ref: 1303245 Nat Genet, 1992
- The rank order of agonist potency, based on inspection of the mean effective concentration values EC50, for IL8RB was GROgamma 1 nM > IL 8 4 nM approximately GROalpha 5 nM approximately GRObeta 4 nM approximately NAP 2  [ **PPBP** ]  7 nM > ENA 78 11 nM, and for  **IL8RA**  was IL 8 4 nM >>> ENA 78 40 nM approximately NAP 2  [ **PPBP** ]  45 nM > GROalpha 63 nM approximately GROgamma 65 nM >> GRObeta.  Ref: 8702798 J Biol Chem, 1996
- CXCR1  [ **IL8RA** ]  and CXCR2 mediate migratory activities in response to IL 8 and other ELR CXC chemokines e.g., GCP 2 and NAP 2  [ **PPBP** ] .  Ref: 12627953 Biochemistry, 2003
- The G protein coupled CXC chemokine receptor CXCR  [ **IL8RA** ]  2 mediates activation of neutrophil effector functions in response to multiple ligands, including IL 8 and neutrophil activating peptide 2 NAP 2  [ **PPBP** ] .  Ref: 10878382 J Immunol, 2000
- J IL8R1  [ **IL8RA** ]  expressed 00 00 copies of IL 8R1, and bound IL 8 with high affinity Kd 1 4 nM and GRO alpha and NAP 2  [ **PPBP** ]  with low affinity Kd 200 500 nM.  Ref: 8137938 FEBS Lett, 1994
- After phagocytosis of opsonized yeast, the expression of CXCR1  [ **IL8RA** ]  and CXCR2 was substantially down regulated and was accompanied by reduced Ca responses to corresponding ligands, IL 8 and neutrophil activating peptide 2 NAP 2  [ **PPBP** ] .  Ref: 12239185 Blood, 2002
- These receptors did not participate in ligand induced receptor trafficking, while surface expressed CXCR  [ **IL8RA** ]  2 was internalized and re expressed following stimulation with NAP 2  [ **PPBP** ] .  Ref: 10878382 J Immunol, 2000

**Non-interactive relationship** (e.g. studied together, co-existance, homology, etc.)

- Investigating the involvement of the two chemokine receptors CXCR 1  [ **IL8RA** ]  and CXCR  [ **IL8RA** ]  2 in NAP 2  [ **PPBP** ]  mediated chemotaxis, we observe that the cells become desensitized to the first optimum of the chemokine after selective downregulation of CXCR  [ **IL8RA** ]  2, while both optima disappear upon simultaneous downregulation of both receptors.  Ref: 9373270 Blood, 1997
- By enzymatic removal of one 9 kDa carbohydrate moiety in surface expressed CXCR  [ **IL8RA** ]  2 we can show that neither NAP 2  [ **PPBP** ]  induced trafficking nor signaling of the receptor is dependent on its full glycosylation.  Ref: 10878382 J Immunol, 2000
- Correspondingly, we found PBP  [ **PPBP** ]  and, less potently, CTAP III to inhibit CXCR  [ **IL8RA** ]  2 but not CXCR 1  [ **IL8RA** ]  dependent chemotaxis of neutrophils toward NAP 2  [ **PPBP** ] .  Ref: 11049972 Blood, 2000
- The differences between IL 8, NAP 2  [ **PPBP** ] , and GRO alpha in their ability to stimulate PLD is likely to be related to their respective binding affinities for the two IL 8 receptors IL 8R A  [ **IL8RA** ]  and IL 8R B.  Ref: 7812007 Blood, 1995
- The differential control of CXCR1  [ **IL8RA** ]  vs CXCR2 internalization by IL 8 and NAP 2  [ **PPBP** ] , as well as by phosphorylation mediated mechanisms, suggests that a chemokine and receptor specific mode of regulation of internalization may contribute to the divergent activities of these receptors.  Ref: 10623425 Cytokine, 1999
- Blocking monoclonal antibodies MoAbs specific for CXCR  [ **IL8RA** ]  2 or CXCR 1  [ **IL8RA** ]  either suppress the first optimum of NAP 2  [ **PPBP** ]  induced chemotaxis or drastically reduce the second one, respectively.  Ref: 9373270 Blood, 1997
- Transendothelial migration of neutrophils in response to CXCL7  [ **PPBP** ]  or IL 8 CXCL8 was subject to modulation by CTAP III, but not CXCL4, as seen by drastic desensitization of the migratory response of neutrophils pre exposed to CTAP III, which was paralleled by selective down modulation of CXCR  [ **IL8RA** ]  2.  Ref: 12193731 J Immunol, 2002
- Two monoclonal antibodies, anti IL8R1  [ **IL8RA** ]  and anti IL8R2, raised against both interleukin 8 receptors IL 8R of human neutrophils, IL 8R1 and IL 8R2, were used to study individual receptor functions after stimulation with IL 8, GRO alpha, or NAP 2  [ **PPBP** ] .  Ref: 8692878 Proc Natl Acad Sci U S A, 1996
- ... IL 8 was 300 to 00 fold more potent than GRO alpha or NAP 2  [ **PPBP** ]  as attractant of J IL8R1  [ **IL8RA** ] .  Ref: 8137938 FEBS Lett, 1994
- To elucidate the role of CXCR1  [ **IL8RA** ]  and CXCR2 in inflammation and to delineate the basis for the divergent regulation of these receptors by IL 8 and NAP 2  [ **PPBP** ] , we characterized the IL 8 and NAP 2  [ **PPBP** ]  induced mechanisms regulating the expression of each receptor, focusing on receptor internalization and recycling.  Ref: 10623425 Cytokine, 1999
- This was evident from experiments in which inhibition of the CTAP III processing protease and simultaneous blockade of the CXCL7  [ **PPBP** ]  high affinity receptor CXCR  [ **IL8RA** ]  2 led to complete abrogation of CTAP III mediated neutrophil adhesion.  Ref: 12193731 J Immunol, 2002
- Thus, proteolytic processing of inactive precursors plays a decisive role in whether the beta TG  [ **PPBP** ]  proteins will act as stimulatory or inhibitory agents in neutrophil activation via the G protein coupled receptors CXCR 1  [ **IL8RA** ]  and 2.  Ref: 11138777 Immunol Rev, 2000
- GROalpha, GRObeta, GROgamma, NAP 2  [ **PPBP** ] , and ENA 78 competed weakly for the high affinity IL 8 binding site at  **IL8RA** .  Ref: 8702798 J Biol Chem, 1996
- Paradoxically,  **IL8RA**  shares 77% amino acid identity with IL8RB but is not a receptor for GRO MGSA or NAP 2  [ **PPBP** ] .  Ref: 8407886 J Biol Chem, 1993
- A functional hierarchy was shown to exist between GCP 2 and 2 other ELR CXC chemokines, IL 8 and NAP 2  [ **PPBP** ] , in their abilities to induce CXCR1  [ **IL8RA** ]  and CXCR2 internalization, according to the following IL 8 > GCP 2 > NAP 2  [ **PPBP** ] .  Ref: 10688807 Blood, 2000
